# CRLF1–MYH9 Interaction Regulates Proliferation and Metastasis of Papillary Thyroid Carcinoma Through the ERK/ETV4 Axis

**DOI:** 10.3389/fendo.2020.00535

**Published:** 2020-08-25

**Authors:** Shi-Tong Yu, Bai-Hui Sun, Jun-Na Ge, Jiao-Long Shi, Man-Sheng Zhu, Zhi-Gang Wei, Ting-Ting Li, Zhi-Cheng Zhang, Wei-Sheng Chen, Shang-Tong Lei

**Affiliations:** Department of General Surgery, Nanfang Hospital, Southern Medical University, Guangzhou, China

**Keywords:** CRLF1, MYH9, EtV4, tumor progression, papillary thyroid carcinoma

## Abstract

In our previous study, we have shown that CRLF1 can promote proliferation and metastasis of papillary thyroid carcinoma (PTC); however, the mechanism is unclear. Herein, we investigated whether the interaction of CRLF1 and MYH9 regulates proliferation and metastasis of PTC cells via the ERK/ETV4 axis. Immunohistochemistry (IHC), qPCR, and Western blotting assays were performed on PTC cells and normal thyroid cells to profile specific target genes. *In vitro* assays and *in vivo* assays were also conducted to examine the molecular mechanism. Results showed that CRLF1 directly bound MYH9 to enhance the stability of CRLF1 protein. Inhibition of MYH9 in PTC cells overexpressing CRLF1 significantly reversed malignant phenotypes, and CRLF1 overexpression activated ERK pathway, *in vitro*, and *in vivo*. RNA-sequencing revealed that ETV4 is a downstream target gene of CRLF1, which was up-regulated following ERK activation. Moreover, it was revealed that ETV4 is highly expressed in PTC tissues and is associated with poor prognosis. Finally, the ChIP assays showed that ETV4 induces the expression of matrix metalloproteinase 1 (MMP1) by binding to its promoter on PTC cells. Altogether, our study demonstrates that CRLF1 interacts with MYH9, promoting cell proliferation and metastasis via the ERK/ETV4 axis in PTC.

## Introduction

Papillary thyroid carcinoma (PTC) contributes nearly 90% of all thyroid malignancies. It is therefore the most common endocrine cancer (1). The prognosis of majority of PTC patients is good; however, a subset of patients with aggressive subtype of PTC have poor prognosis (2). Accumulating evidence reveals that mutations, BRAF^V600E^ and TERT promoter, activate mitogen-activated protein kinase (MAPK/ERK) and phosphoinositide 3-kinase (PI3K)/protein kinase B (AKT) pathways and, therefore, play a crucial role in PTC (3–7). Currently, the pathogenesis of PTC is not fully understood.

The Cytokine Receptor-Like Factor 1 (CRLF1) promotes proliferation and survival of normal neuron cells and B-cells by assembling with the cardiotrophin-like cytokine factor 1 (CLCF1) or p28. Its effects are mediated through the MAPK/ERK and PI3K/AKT pathways (8, 9). In PTC, CRLF1 enhances proliferation and invasion of PTC cells and activates the MAPK/ERK and PI3K/AKT pathways in *in vitro* and *in vivo* settings (10). However, it is currently not fully understood how CRLF1 regulates malignant phenotypes of PTC.

The *MYH9* gene codes for the non-muscle myosin heavy-chain IIA protein (a cytoskeletal protein) that regulates several biological activities by interacting with other genes, including contraction, cell migration, polarity formation, the formation of focal adhesions, and the epithelial–mesenchymal transition (EMT) (11). In gastric and colorectal cancers, MYH9 accelerates tumor development by enhancing their invasion and metastatic ability (12, 13). Inconsistently, a study reported that *MHY9* gene might act as a tumor suppressor in the head and neck cancers (14). Although MYH9 binds to lncRNA gene PTCSC2 and regulates FOXE1 in the 9q22 thyroid cancer risk locus (15), mechanistically, the detailed role of MYH9 in PTC is still unknown.

The expression of ETS family proteins is regulated by several transcription factors, some of which are substrates of ERK1/2, modulating the expressions of matrix metalloproteases (MMPs) and BCL2 family influences the migration and invasion, and survival of cells (16). ETV4 belongs to the PEA3 subfamily, which is known to promote tumor progression in several cancer types (17, 18). Its role in PTC deserves further research.

Herein, we identified that MYH9 directly binds with CRLF1 and increasing CRLF1 protein stability. Inhibition of MYH9 in PTC cells overexpressing CRLF1 significantly reversed malignant phenotypes, and CRLF1 overexpression activated ERK pathway. RNA-sequencing revealed that ETV4 is a downstream target gene of CRLF1, which was up-regulated following ERK activation. Moreover, ETV4 is highly expressed in PTC tissues and is associated with poor prognosis. Finally, the chromatin immunoprecipitation (ChIP) assays showed that ETV4 binds to the promoter of matrix metalloproteinase 1 (MMP1) on PTC cells. Based on these findings, our study demonstrates that CRLF1 interacts with MYH9, promoting cell proliferation and metastasis via the ERK/ETV4 axis in PTC.

## Results

### CRLF1 Interacts With MYH9 and Promotes CRLF1 Protein Stability

**Figure 1:**
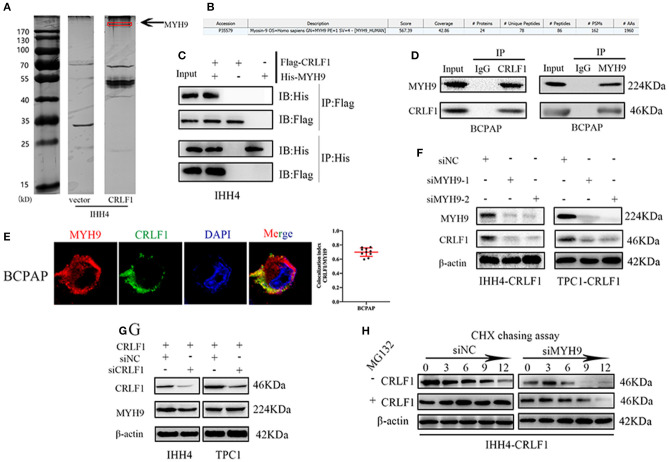
Interaction between CRLF1 and MYH9 in PTC cells. **(A)** Silver staining showing proteins interacting with CRLF1 in IHH4 cells and the molecular weights of MYH9. **(B)** Mass spectrometry analysis reveals MYH9 as a potential binding partner of CRLF1. **(C)** Co-IP showing interaction between exogenous CRLF1 and MYH9 in IHH4 cells. **(D)** Co-IP experiments reveal the interaction between endogenous CRLF1 and MYH9 in BCPAP cells. **(E)** Immunofluorescence staining showing the cytosolic colocalization of CRLF1 protein and MYH9 protein in PTC cells. Colocalization analysis was performed in single cells with JACoP plugin in ImageJ. **(F)** MYH9 and CRLF1 protein expression after MYH9 knockdown by siRNA approach as detected by Western blotting assay. **(G)** MYH9 and CRLF1 protein expression after CRLF1 overexpression or knockdown as detected by Western blotting assay. **(H)** Western blot results showing the effect of MYH9 knockdown on CRLF1 expression in PTC cells treated with cycloheximide at different time points and in the presence of MG132. β-Actin served as the loading control.

In the previous study (10), we examined CRLF1 expression level in normal and PTC cell lines, and used lentiviral vector to overexpress CRLF1 in the PTC cell lines IHH4 and TPC1 (Figures S1A,B). Next, coimmunoprecipitation (Co-IP), silver staining, and mass spectrometry were carried out in IHH4-CRLF1 cells to identify a binding protein of CRLF1. Consequently, several CRLF1-interacting proteins were identified, among which MYH9 (224-kDa band) had the highest match score (Figures 1A,B). Further analysis revealed that CRLF1 interacted with MYH9 in PTC cells as evidenced by exogenous and endogenous Co-IP (Figures 1C,D and Figure S1C). Moreover, immunofluorescence revealed that CRLF1 and MYH9 proteins colocalized in the cytoplasm of PTC cells (Figure 1E and Figure S1D). These results indicated that CRLF1 interacted with MYH9 in PTC cells.

Further, suppression of MYH9 markedly decreased the levels of the CRLF1 protein (Figure 1F), while CRLF1 had no impact on MYH9 expression (Figure 1G). Additionally, using the qPCR assay, we could not establish any connection between CRLF1 and MYH9 at the mRNA level (Figures S2A,B). Similarly, the bioinformatics analyses of the TCGA data revealed no marked correlation between CRLF1 and MYH9 at the mRNA level (Figure S2C).

The cycloheximide (CHX) chasing assay revealed that MYH9 enhanced the half-life of the CRLF1 protein. The impact of MYH9 on CRLF1 stability was attenuated by MG132, the proteasome inhibitor (Figure 1H). Altogether, these findings indicated that CRLF1 interacts with MYH9, therefore, increasing its protein stability.

### MYH9 Knockdown Reverses CRLF1-Induced Tumor Proliferation on PTC Cells

Firstly, we examined MYH9-mRNA expression levels using the TCGA-database samples. Our analyses revealed that MYH9 expression levels were not significantly associated with PTC or the late clinical stage (Figures S2D,E). These findings suggest that MYH9 alone does not play a crucial role in PTC.

**Figure 2:**
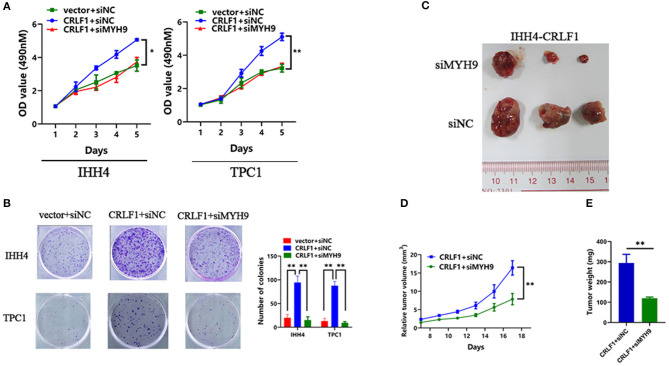
Effects of MYH9 knockdown on CRLF1-induced growth of PTC cells. **(A)** MYH9 knockdown significantly alleviated the effects CRLF1 on cell viability in IHH4 and TPC1 cells. Data are presented as the mean ± SD. **(B)** MYH9 knockdown significantly reversed the effects of CRLF1 on colony formation in IHH4 and TPC1 cells. Data are presented as the mean ± SD. **(C)** Three representative tumors from siMYH9- and siNC-transfected IHH4 cells overexpressing CRLF1 (IHH4-CRLF1) in nude mice. **(D)** Tumor growth curves of siMYH9-transfected IHH4 cells overexpressing CRLF1in nude mice compared with those of siNC-transfected control cells. Data are presented as the mean ± SD. **(E)** Histogram showing the mean tumor weights from the CRLF1+siMYH9 group and the CRLF1+siNC group. Data are presented as the mean ± SD. Significant differences are indicated as follows: *P < 0.05 and **P < 0.01.

Further, rescue experiments were performed to determine the potential impact of MYH9 on the effects of CRLF1 on malignant phenotypes in IHH4 cells. We transfected two siRNAs-MYH9 into CRLF1-overexpressing and empty vector IHH4 cells. Consequently, the qPCR and Western blot assays validated that MYH9-mRNA and MYH9-protein levels declined (Figures S3A,B). In IHH4 cells transfected with vector, knocking down MYH9 did not affect cell proliferation and colony formation ability (Figures S3C,D). Notably, in CRLF1-overexpressed IHH4 cells, interfering MYH9 decreased cell proliferation and colony formation ability significantly (Figures 2A,B). Further, we also examined whether knocking down MYH9 in CRLF1-overexpressing TPC1 cells would affect tumor proliferation and colony formation. These results were consistent with those in IHH4 cells (Figures 2A,B).

Next, we evaluated whether MYH9 reverses CRLF1-induced tumor proliferation *in vivo*. Knocking down MYH9 in CRLF1-overexpressing IHH4 cells markedly lowered tumor formation with significantly smaller volumes of tumors reported compared with the cells transfected with siNC (Figures 2C,D). Consequently, we dissected the xenograft tumors and weighed. The results indicated that the average tumor weight in the siMYH9 group was significantly lower than in the siNC group (*P* < 0.05, Figure 2E).

### MYH9 Knockdown Reverses CRLF1-Induced Tumor Metastasis and EMT on PTC Cells

**Figure 3:**
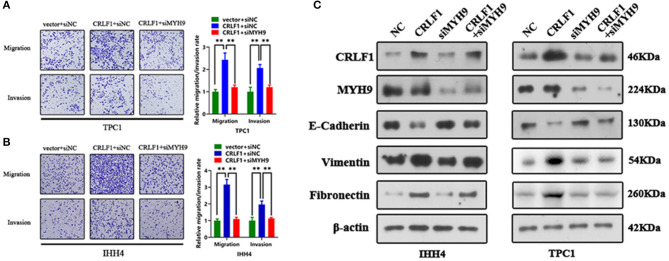
Impact of MYH9 knockdown on the effects of CRLF1 on the migration, invasion, and EMT process of PTC cells. **(A)** Representative images showing the migrating/invading TPC1 cells expressing the vector or CRLF1 plasmid, which were then transfected with siNC or siMYH9. The histograms show the mean ± SD of the number of migrating/invading cells from three independent assays. **(B)** Representative images showing the migrating/invading IHH4 cells expressing the vector or CRLF1 plasmid, which were then transfected with siNC or siMYH9. The histograms show the mean ± SD of the number of migrating/invading cells from three independent assays. **(C)** Protein levels of EMT markers that changed following CRLF1 knockdown in PTC cell lines overexpressing CRLF1. β-Actin served as the loading control. Significant differences are indicated as follows: **P < 0.01.

Inhibited MYH9 in PTC cells transfected with empty vector had no impact on the tumor migration and invasion ability (Figure S4A). The PTC cells transfected with si-MYH9 had a significantly lower number of migrated cells compared with the siNC group. Similarly, the invasion assays revealed that decreased MYH9 in CRLF1-overexpressing PTC cells resulted in a lower number of cells invading via the Matrigel-coated membrane (Figures 3A,B). These results point out that CRLF1 promotes cell migration and invasion ability depending on MYH9.

Epithelial–mesenchymal transition (EMT) is an essential biological process in cancer metastasis. Previously, we had shown that CRLF1 induced PTC cell metastasis by activating the EMT process (10). Therefore, herein, we examined the connection between EMT markers in CRLF1-overexpressing PTC cells and the knocking down MYH9. Consequently, the levels of the epithelial marker E-cadherin were markedly increased (Figure 3C). However, the mesenchymal markers, fibronectin, and vimentin were significantly decreased in cells transfected with siMYH9 compared with the cells transfected with NC. We also identified change in cell morphology (Figure S4B). Altogether, these results imply that knocking down MYH9 reverses CRLF1-induced tumor metastasis and EMT in PTC cells.

### MYH9 Knockdown Antagonizes the ERK Pathway Activated by CRLF1

Previous evidence revealed that CRLF1 activates the ERK pathway in PTC cells (10). Therefore, to further elucidate the impact of MYH9 on CRLF1-modulated pathways, siMYH9 was transiently transfected into CRLF1-overexpressing PTC cells. The findings indicated that the siMYH9-transfected CRLF1-overexpressing PTC cells had reduced ERK1/2 phosphorylation levels compared with the control cells (Figure S5A). We also examined p-ERK phosphorylation in tumors generated from CRLF1-overexpressing IHH4 cells. Reduced levels of p-ERK phosphorylation were detected in siMYH9-transfected cells compared with the siNC-transfected cells (Figure S5B). Collectively, CRLF1 interacts with MYH9 promoting PTC malignant phenotypes via the MAPK/ERK pathway activation.

### RNA-Sequence Reveals That ETV4 Is a Downstream Target of CRLF1

**Figure 4:**
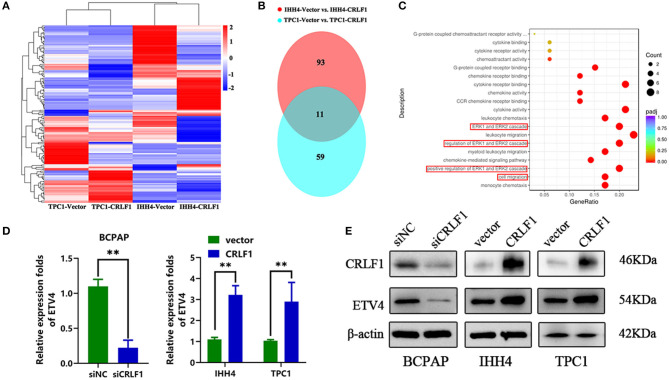
ETV4 is the downstream target gene of CRLF1 in PTC. **(A)** After CRLF1 overexpression, the downstream mRNA sequence was analyzed. **(B)** A Venn diagram showing 11 co-altered genes in the two groups of PTC cells. **(C)** Bioinformatic analysis showing a significant association between ERK pathway and cell migration after CRLF1 overexpression. **(D)** The mRNA expression level of ETV4 after CRLF1 knockdown or overexpression in PTC cells as measured by qPCR analysis. **(E)** The protein expression level of ETV4 after CRLF1 knockdown or overexpression in PTC cells as measured by Western blot assay. β-Actin served as internal reference gene for the qPCR assays and as a loading control for the Western blot assays. Data are presented as the mean ± SD. Significant differences are indicated: **P < 0.01.

We performed RNA-Sequencing (RNA-Seq) on IHH4 and CRLF1-overexpressing TPC1 cells to determine the genes controlled simultaneously by CRLF1 (the heat maps are shown in Figure 4A). We used a Venn diagram to show the results of the investigation of the potential downstream genes of CRLF1 (Figure 4B). The up-regulation of CRLF1 in IHH4 cells caused differential expression of 94 genes, up-regulation of 33 genes, and down-regulation of 61 genes. Moreover, the CRLF1's up-regulation in TPC1 cells altered the expression of 59 genes, up-regulation of 30 genes, and down-regulation of 29 genes. Combining the deregulated genes from the two groups, we identified 11 consistently altered genes. Enrichment analysis of the merged–altered genes revealed that the genes were enhanced in the cell migration pathways as well as the MAPK/ERK pathways (Figure 4C). Notably, ETV4 was the highly statistically significant gene (Table S1). Previously, evidence revealed that ETV4 is regulated by the activation of the ERK pathway vial sumoylation, ubiquitination, and p300-mediated acetylation. To validate these findings, we used qPCR and Western blot assays to examine ETV4 mRNA and protein expression levels. The findings indicated that knocking down CRLF1 in BCPAP cells reduces ETV4 mRNA and protein levels, while overexpressing CRLF1 increases both ETV4 mRNA and protein levels (Figures 4D,E). Altogether, these results suggest that ETV4 is a prospective downstream gene of CRLF1.

### ETV4 May Promote Tumorigenesis by Binding to the MMP1 Promoter

ETV4, an ETS family protein, is a transcription factor that functions as substrates for ERK1/2 (16). Research shows that ETV4 is associated with tumor progression in several cancer types; in thyroid cancer, it may bind to the mutated TERT promoter, therefore increasing TERT expression (19). However, the expression of ETV4 in PTC remains unclear.

**Figure 5:**
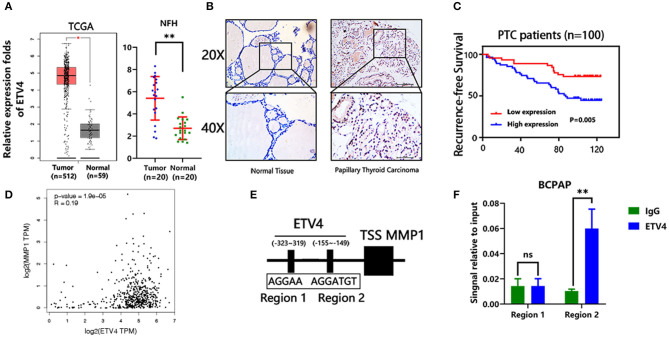
ETV4 transcriptionally up-regulates MMP1 expression and correlates with PTC progression. **(A)** The mRNA expression level of ETV4 in the tumor (*n* = 512) and normal (*n* = 59) thyroid tissues from TCGA cohort (analysis generated from Gepia: http://gepia.cancer-pku.cn/). The mRNA expression level of ETV4 in the tumor (*n* = 20) and normal (*n* = 20) thyroid tissues from the NFH (Nanfang Hospital) cohort. **(B)** Representative IHC staining for ETV4 in PTC and normal tissue. ETV4 is mainly localized in PTC cell nucleus. **(C)** Kaplan–Meier survival analysis showing that higher ETV4 expression levels are significantly associated with poor recurrence-free survival in all PTC patients (*P* = 0.005). **(D)** The association between mRNA expression level of ETV4 and MMP1 in the TCGA database (analysis generated from Gepia: http://gepia.cancer-pku.cn/). **(E)** Bioinformatic prediction of the binding sites of ETV4 within the promoter region of MMP1. **(F)** Chromatin immunoprecipitation analysis (all groups vs. IgG group) (*n* = 3 independent experiments, Student's *t*-test) of ETV4 binding to the MMP1 promoter. **P < 0.01.

In the mRNA expression profile, ETV4 was markedly higher in PTC tissues than in the normal thyroid tissue both in the TCGA and NFH cohort (Figure 5A). Next, we performed the IHC analyses of ETV4 using the 100 paraffin-embedded PTC samples. Consequently, ETV4 was primarily localized in the PTC cell nucleus (Figure 5B). Table 1 shows a summary of the clinical characteristics of PTC patients. No substantial differences in the association were found between ETV4 expression and age, sex, and T classification. However, we identified that a higher ETV4 expression level was associated with lymph node metastasis, recurrence, distant metastasis, and the TNM stage (*P* < 0.05). Additionally, we found out that PTC patients with elevated levels of ETV4 had a shorter recurrence-free survival time than those with low ETV4 expression (log-rank test, *P* < 0.05, Figure 5C).

**Table 1:** The clinicopathological parameters in 100 PTC patients.

**Variables**	**All cases (*n* = 100)**	**ETV4**		***P-*value**
		**Low expression (*n* = 45) (%)**	**High expression (*n* = 55) (%)**	
**Sex**				
Male	29	12 (26.7%)	17 (30.9%)	0.64
Female	71	33 (73.3%)	38 (69.1%)	
**Age**				
<55	78	39 (86.7%)	39 (70.9%)	0.09
≥55	22	6 (13.3%)	16 (29.1%)	
**T classification**				
T1+T2	62	29 (64.4%)	33 (60.0%)	0.65
T3+T4	38	16 (35.5%)	22 (40.0%)	
**Lymph node metastasis**				
Absent (N0)	42	24 (53.3%)	18 (32.7%)	0.03^*^
Present (N1)	58	21 (46.7%)	37 (67.3%)	
**Distant metastasis**				0.02^*^
Absent (M0)	83	42 (93.3%)	41 (74.5%)	
Present (M1)	17	3 (6.7%)	14 (25.5%)	
**TNM stage**				0.04^*^
I+II	80	40 (88.9%)	40 (72.8%)	
III+IV	20	5 (11.1%)	15 (27.3%)	
**Recurrence**				<0.01^*^
No	60	34 (75.6%)	26 (47.3%)	
Yes	40	11 (24.4%)	29 (52.7%)	

* *P < 0.05. The bold indicates p-value with statistical significance*.

Next, we explored the potential mechanism through which ETV4 modulates PTC proliferation and metastasis. In liver and gastric cancer types, ETV4 positively modulates MMP1 by binding the MMP1 promoter regions (17). Moreover, MMP1 is known to play a crucial role in many cancers' progression, including thyroid cancer (20). First, we found out that there was a marked association between ETV4 and MMP1 in mRNA expression level in the TCGA cohort (*R* = 0.19, *p* = 1.9 × e^−5^, Figure 5D). We used the JASPAR (http://jaspar.genereg.net) database analysis to predict the potential binding site of ETV4 to the MMP1 promoter (Figure 5E). In the ChIP-PCR analysis, the ETV4 antibody and primer sets recognizing binding regions 1–2 revealed that, in BCPAP cells, ETV4 markedly interacted with region 2 and not region 1 of the human MMP1 proximal promoter (Figure 5F). Compared with binding IgG only, the interaction was strong and substantial. There was enrichment in the ETV4 antibody group compared with IgG in binding regions that contained ETV4 binding sequences at bp −155 to −149 (5′-AGGTGT-3′). Altogether, these findings indicated that ETV4 directly regulates the MMP1 expression; therefore, we speculated that ETV4 may increase PTC cell proliferation and metastasis abilities.

**Figure 6:**
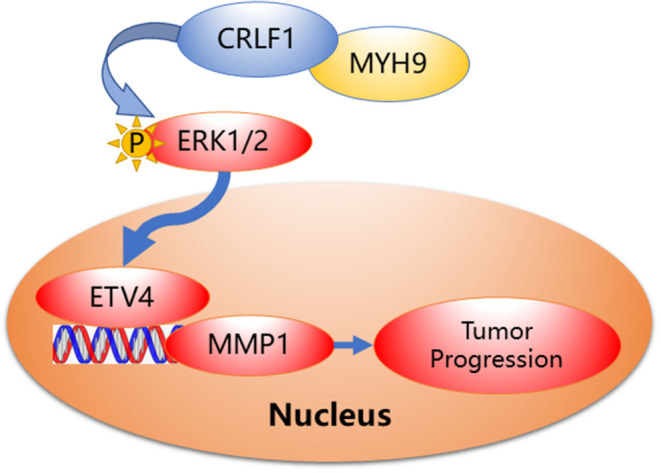
Proposed model demonstrating interactions between CRLF1, MYH9, and activation of the ERK/ETV4 axis to regulate PTC progression.

Collectively, based on our findings, we speculate a simple model to elucidate the molecular mechanism of CRLF1 interaction with MYH9, promoting progression in PTC (Figure 6). In brief, CRLF1 interacts with MYH9 and activates the ERK pathway, thereby up-regulating downstream target ETV4. ETV4 transcriptionally activates its downstream gene, MMP1, therefore promoting PTC cell proliferation, migration, and invasion.

## Discussion

Herein, we firstly demonstrated that CRLF1 interacts with MYH9 inducing PTC cell proliferation and metastasis through the ERK/ETV4 pathway, both *in vitro* and *in vivo*. Previously, we had revealed that CRLF1 promotes cell proliferation and metastasis in PTC cells (10). However, the molecular mechanism is still unknown.

We used the FLAG pull-down assay and mass spectrometry to evaluate MYH9 as the prospective interacting protein with CRLF1 in PTC cells to elucidate further the detailed molecular mechanism of CRLF1 as a promoter of malignant phenotypes. We verified these findings using the Co-IP assays. Previous studies indicated that the MYH9 performs diverse functions in different cancer types (12–14). In PTC, MYH9 binds the lncRNA gene, PTCSC2, to modulate FOXE1 in the 9q22 thyroid cancer risk locus (15). However, no research in available literature has explored the function of MYH9 by manipulating its expression levels in PTC cells. In this study, we first established that there is no association between MYH9 expression and PTC progression using the TCGA cohort alone. Therefore, we speculated that MYH9 interacts with a second molecule to enhance PTC progression. Consequently, we found out that the suppression of MYH9 decreased CRLF1 protein levels. However, CRLF1 did not regulate MYH9 expression. Further, the CHX chasing assays revealed that CRLF1 interacts with MYH9 by enhancing its protein stability.

Next, the rescue experiments confirmed that interfering with MYH9 significantly reduces tumor proliferation on CRLF1-overexpressing PTC cells but not in vector-expressing cells, *in vitro*, and *in vivo*. Since tumor invasion and metastasis are the leading cause of cancer-related death, including thyroid cancer, we tested the effect of MYH9 on PTC cell migration and invasion. Consequently, down-regulating MYH9 in the CRLF1-overexpressing PTC cells decreased cell migration and invasion significantly but not in the vector-expressing PTC cells. EMT is an essential process in tumor transformation cascade, which is associated with cancer progression and metastasis (21). In this study, our findings showed that interfering with MYH9 expression in PTC cells up-regulates the epithelial marker (E-cadherin) and down-regulates mesenchymal markers (fibronectin and vimentin) in CRLF1-overexpressing IHH4 and TPC1 cells. These results suggested that CRLF1-induced PTC cell malignant phenotypes are MYH9-dependent.

The ERK pathway plays a vital part in the pathogenesis, as well as the progression of PTC (22). In our previous study, we revealed that CRLF1 activates the ERK pathway in PTC (10). In the present study, interfering with MYH9 de-activated p-ERK in CRLF1-overexpressing PTC cells. The p-ERK expression was decreased in xenograft tumors generated from siMYH9-transfected cells compared to those transfected with siNC. These findings indicated that CRLF1 interacts with MYH9 to activate the ERK pathway.

We additionally demonstrated that ETV4 is the downstream gene of CRLF1 in PTC. ETV4 is regulated by sumoylation, ubiquitination, and p300-mediated acetylation through ERK activation (23, 24). Recently, it was reported that ETV4 selectively binds to the mutant TERT promoter and up-regulates its expression in PTC cells (19). Herein, for the first time, we confirmed that ETV4 expression level was higher in PTC tissues and is associated with poor prognosis from clinical perspectives; these data indicated that ETV4 may serve as a therapeutic target for PTC patients. Our results additionally showed that ETV4 induces tumor growth, cell migration, and the EMT process by interfering ETV4 expression in PTC cells. Moreover, we provide evidence that MMP1 is a crucial downstream target gene of ETV4 in PTC through the ChIP assays. MMP1 has been reported to function as an oncogene in various cancer types (20), including thyroid cancer (25–28). Notably, most MMP genes possess ETS binding elements in their promoter (29). Recently, it was shown that ETV4 binds to the MMP1 promoter in HCC (18), consistent with our findings. However, the final effects of MMP1 was not validated, which added a limitation to the current study. Additional experiments, such as zymography and so on, to further validating its functions in the PTC are guaranteed in future studies. Therefore, we evidentially show that ETV4 promotes tumor progression by transcriptionally regulating MMP1 in PTC cells.

Altogether, our findings elucidate the molecular mechanism of CRLF in inducing malignant phenotypes in PTC. We demonstrated that CRLF1 interacts with MYH9, promoting tumor proliferation and metastasis by activating ERK/ETV4; therefore, we provide a prospective therapeutic target for the treatment of PTC.

## Materials and Methods

### Ethical Statement

We conducted all the animal experiments following a protocol approved by the Animal Care and Use Committee of the Southern Medical University. We obtained written consent from all patient-subjects for the use of clinical materials for research purposes. Besides, we obtained ethical approval of this study from the Ethics Committee of the Nanfang Hospital.

### Cell Culture

Prof. Haixia Guan (The First Affiliated Hospital of China Medical University, Shenyang, China) helped us with the human PTC IHH4, BCPAP, and normal thyroid epithelial Nthy-ori-3-1 cell lines. We purchased the human PTC cell line TPC-1 and the human embryonic kidney 293T (293T) cell lines from Nanjing Cobioer Company (Cobioer, China) and the American Type Culture Collection (ATCC, Manassas, VA, USA), respectively. We cultured the IHH4 cell line in RPMI-1640 and Dulbecco's modified Eagle's medium (DMEM; Invitrogen) enriched with 10% fetal bovine serum (FBS, Gibco, USA). Next, we cultured the Nthy-ori-3-1 and BCPAP cell lines in RPMI-1640 augmented with 10% FBS. We cultured the TPC-1 and 293T cell lines in DMEM supplemented with 10% FBS. Notably, we cultured all the cell lines with penicillin (100 U/ml) and streptomycin (100 U/ml) at 37°C in a humidified 5% CO2 incubator.

### Clinical Tissues and Specimen

We obtained 20 PTC tissue samples and their paired normal tissue samples in August 2018 for the qRT-PCR assays. We collected a total of 100 paraffin-embedded PTC samples from patients who were first diagnosed between January 2005 and December 2009 at Nanfang Hospital, Southern Medical University, for the IHC assays (30, 31). We re-documented all the medical history of the participants as per the 8th Edition of the American Joint Committee on Cancer (AJCC) TNM system. We obtained these samples from 39 men and 162 women with a median age of 41 years (range, 14–74). We followed up all the patients every 3–5 months during the first 5 years and then every year after that. Recurrence disease referred to as recurrent with structural diseases. We defined the patients with proven histology or cytology results or suspicious lesions according to imaging studies as having structural disease (10). We defined recurrence-free survival as the time from the date of surgery to the date of relapse, metastasis, or the last follow-up. All patients' survival statuses were confirmed in December 2019.

### Western Blot Analysis

We lysed the total protein in one sodium dodecyl sulfate (SDS) sample buffer and used BCA protein assays to determine protein concentrations. We used 8–12% SDS-polyacrylamide gels by electrophoresis to separate the protein extracts and transferred them to polyvinylidene fluoride membranes (Millipore, USA). Subsequently, we used 5% skim milk or bovine serum albumin to block the proteins for 1 h. Then, we incubated the membranes with primary antibodies at 4°C overnight and then with horseradish peroxidase-conjugated secondary antibodies (Pierce, USA) at room temperature for 1 h. Next, we visualized the bound antibodies through enhanced chemiluminescence and captured using a XAR film. We used β-actin as a loading control.

The primary antibodies used in this study included human anti-CRLF1 (1:400; ab56500), anti-MYH9 (1:1000; ab55456), anti-MMP1 (1:1000; ab215979), anti-fibronectin (1:1000; ab32419), anti-vimentin (1:1000; ab8978), and anti-snail (1:1000; ab216347) from Abcam, USA; anti-ETV4 (1:1000; SAB1403795) and β-actin (1:4000, A5541) from Sigma-Aldrich, USA; and anti-E-cadherin (1:1000; #14472), anti-ERK1/2 (1:1000; #4695), and anti-p-ERK1/2 (1:1000; #4370) from Cell Signaling Technology, USA. All the blot figures included the location of molecular size markers.

### qPCR Assay

We used the TRIzol Reagent (Invitrogen, USA) in isolating total RNA from the PTC cells and clinical tissues following the manufacturer's protocol. After that, we reverse-transcribed 2 μg of RNA using the M-MLV Reverse Transcriptase kit (Promega) as per the manufacturer's instructions. We established the threshold cycle value for each sample by qPCR using SYBR Green (Invitrogen) and a CFX96 Touch sequence detection system (Bio-Rad, USA). We used the β-actin gene as the internal control for all genes. Subsequently, we calculated the relative gene expression levels using the comparative threshold cycle (2^−ΔΔ*CT*^) method. All experiments were run independently in triplicate, and the sequences of the primers are shown in Table S2.

### RNA Interference and Plasmid Transfection

We bought the effective siRNA oligonucleotides targeting the CRLF1 from Guangzhou Ribobio Company (Guangzhou, China) and transfected using Lipofectamine RNAiMax (Invitrogen) according to the manufacturer's protocol. We purchased the lentiviral vector encoding FLAG-tagged CRLF1 (EX-N0027-Lv121), His-tagged MYH9 (EX-T1335-Lv128), the control vector (EX-EGFP-Lv105), and the packaging system (HIV) from GeneCopeia (USA). Subsequently, we verified all the plasmids via DNA sequencing. The siRNA sequences used are shown in Table S3. We co-transfected the lentivirus packaging expression plasmids into the 293T cells. Then, we collected the supernatants containing viruses and used them to infect the PTC cell lines for 48 h. After that, we selected the stable clones of these cells using puromycin (Sigma-Aldrich, USA) for 7 days after infection. We used qRT-PCR and blotting assays to assay for the expression levels of CRLF1 and MYH9 western.

### MTT and Colony Formation Assays

We seeded a total of 600–800 cells in 200 μl of medium per well in 96-well plates (five replicates of each sample). Next, we added 20 μl of MTT (5 mg/ml, BD Biosciences) to each well on the indicated day (days 1, 2, 3, 4, or 5) and incubated for 4 h at 37°C. Then, we discarded the supernatants and added 100 μl of dimethylsulfoxide (DMSO) to each well to dissolve the crystals. After that, we used the spectrophotometric plate reader (BioTek ELX 800, USA) to measure the absorbance at 490 nm. For the colony formation assays, we seeded 800 cells in 2 ml of the medium into each well of a six-well plate and cultured for 7–10 days. We subsequently fixed the colonies with methanol for 10 min and stained with 0.5% crystal violet for 15 min. ImageJ software was used to measure the cell counts. We performed the experiments in triplicate.

### Transwell Assays

We used Transwell chambers (8 μm pores, Corning, USA) for the cell migration and invasion assays. We first pre-coated without (migration assay) or with (invasion assay) Matrigel (BD Biosciences). After that, we seeded 5 × 10^4^-5 × 10^5^ cells suspended in 200 μl of a serum-free medium in the upper chambers, and plated 500 μl of medium augmented with 10% FBS in the lower chambers. After 48 h of incubation, we fixed the cells on the upper surface of the membrane with methanol for 10 min and stained with 0.5% crystal violet for 10 min. Subsequently, we used an inverted microscope to count the cell numbers.

### Animal Studies

The Institutional Research Medical Ethics Committee of Nafang Hospital approved all the animal experiments. We purchased 4- to 6-week-old female BALB/c nude mice (*n* = 6) from Beijing Vital River Laboratory Animal Technology Co., Ltd. (Beijing, China) and randomly divided them into two groups (three mice per group). After that, we established tumor xenografts by subcutaneously injecting 100 μl of a mixture containing 70% siNC-transfected or siMYH9-transfected CRLF1-overexpressing IHH4 cells (1 × 10^6^) and 30% Matrigel. Tumor sizes were measured every 2 days and tumor volumes were calculated using the following equation: 0.5 × length × width^2^. After 17 days, the mice were sacrificed, and the tumors were harvested, weighed, and embedded in 10% paraffin. Each tissue was used to analyze the expression of markers (MYH9, p-ERK1/2) by IHC, as described previously.

### IHC Analysis

We embedded the clinical PTC tissue samples and tumors resected from mice in paraffin. Briefly, we cut 4-m-thick sections and baked them at 60°C for 2 h. Then, we deparaffinized the sections with xylene and rehydrated and blocked the endogenous peroxidase activity with 0.3% H_2_O_2_. Next, we processed the sections for high-temperature antigen retrieval with citrate (pH 6.0) and incubated them with 5% bovine serum albumin to block nonspecific binding. After that, we incubated the sections with diluted rabbit anti-ETV4 antibody (1:100; SAB1403795, Sigma-Aldrich, USA), p-ERK1/2 antibody (1:400; #4370, Cell Signaling Technology, USA), or MYH9 (1:100; ab55456, Abcam, USA) at 4°C overnight. Next, we washed the slides thrice with phosphate-buffered saline plus 1:1000 Tween-20 and incubated them with secondary antibodies (1:1000) for 30 min at 37°C. Then, we immersed slides in diaminobenzidine (Zhongshan Biological and Technical Company, Beijing, China) for 10 min, and then terminated reactions using distilled water. Next, we counterstained the slides with hematoxylin, dehydrated, and cover-slipped. Two experienced pathologists scored all sections. We calculated the staining index of ETV4 as follows: staining index = staining × intensity proportion of positive tumor cells. We defined the staining intensity as follows: 0 (no staining); 1 (weak, light yellow); 2 (moderate, yellow-brown); and 3 (strong, brown). We defined the percentage of positive cells as follows: 0 (no positive cells); 1 (<10% positive tumor cells); 2 (10–50% positive tumor cells); and 3 (>50% positive tumor cells). We determined the staining index cutoff value for ETV4 expression using its median value (3 points). We used a staining index score of >3 points and 3 points to define the tumors with high expression, low expression, respectively.

### FLAG Pull-Down Assay and Silver Staining

We transfected 293FT cells with an Lv-105 empty vector or the Lv121-CRLF1-flag plasmid. Thirty-six hours later, we treated the cells with IP lysis buffer (150 mM NaCl; 1% NP-40) on ice for 30 min. We then collected the proteins and centrifuged them to discard any precipitates. Then, we added 30 μl of FLAG beads (Sigma) to each sample and mixed them at 4°C overnight. The next day, we centrifuged and discarded the supernatant. We washed the beads five times with the IP buffer. After that, we added 30 μl of FLAG peptide (200 μg/ml, Sigma), and the mixture was shaken for 2 h. Next, we centrifuged the supernatant, followed by denaturing, and then performed SDS-PAGE. After electrophoresis, the gel was released and fixed for an hour and then washed with 30% ethanol for 10 min followed by a sensitizer for 2 min. After washing the gel with ultrapure water twice, we added a silver solution for 10 min. Subsequently, we performed de-staining and terminated the reaction when the expected bands appeared. The protein bands present were subsequently used for liquid chromatography–mass spectrometry (LC-MS) analysis.

### Co-Immunoprecipitation

We lysed the cell lines in a protein lysis buffer [20 mM Tris–HCl (pH 7.5), 150 mM NaCl, 1 mM Na_2_EDTA, 1 mM EGTA, 1% Nonidet P-40, 1% sodium deoxycholate, 2.5 mM sodium pyrophosphate, 1 mM β-glycerophosphate, 1 mM Na_3_VO_4_, and 1 μg/ml leupeptin with protease inhibitor cocktail and phosphatase inhibitors] at 4°C for 30 min and centrifuged at 12,000 rpm (10 min, 4°C) to remove cell debris. For immunoprecipitation, we added the antibodies against FLAG or His to the lysates and incubated them overnight at 4°C, with rabbit IgG (1:100) serving as a control antibody. Then, we added the protein A/G agarose beads and incubated them with the lysates for 1 h at 4°C. After that, we washed the beads with the protein lysis buffer five times and any remaining bound proteins were eluted in protein loading buffer and analyzed by immunoblotting.

### Immunofluorescence and Confocal Microscopy

We plated the cells on coverslips in 48-well plates and cultured them overnight to allow for cell adherence. After fixation with 4% paraformaldehyde and permeabilization with 0.2% Triton X-100, we incubated the cells with antibodies. IgG was added as negative control. After that, we counterstained the cells with 0.2 mg/ml DAPI (Sigma-Aldrich, USA) and visualized them with a fluorescent confocal microscope (Carl Zeiss LS800, Germany). We used primary antibodies, including MYH9 (1:50; ab55456) and CRLF1 (1:50; ab56500) from Abcam, USA. Colocalization analysis was performed in single cells with JACoP plugin in ImageJ. Pearson's P colocalization index was calculated.

### RNA-Seq and Bioinformatic Analysis

RNA was quantified in a Nanodrop (Thermo Scientific, Waltham, MA, USA) and quality checked using a Bioanalyzer-RNA 6000 nano kit (Agilent Technologies, Santa Clara, CA, USA). We prepared the libraries from 1 μg of RNA using the TruSeq Stranded mRNA kit (Illumina, San Diego, CA, USA). We performed Next-generation sequencing a NextSeq 500 platform (Illumina, San Diego, CA, USA) and a minimum of 30 million reads for each replicate were generated. The Cufflink RNA-Seq workflow was employed to perform bioinformatics analysis. We calculated differential gene expression as a log2 fold change (vector/CRLF1). The optimized FDR approach was employed to adjust *p*-values of the differentially expressed (FDR cutoff = 0.05) and genes with adjusted *p*-value (*q* value) <0.05 were considered significantly regulated.

### The Chromatin Immunoprecipitation (ChIP) Assay

The ChIP assay was carried out using a ChIP assay kit (Thermo Scientific, Waltham, MA, USA). All procedures were performed according to the manufacturer's protocol. Briefly, chromatins were crosslinked, isolated, and digested with Micrococcal Nuclease to obtain DNA fragments. The samples were treated with the antibody or IgG for immunoprecipitation. After elution and purification, the recovered DNA fragments were subjected to qPCR and PCR assays. IgG served as a negative control.

### The Cycloheximide (CHX) Chase Assay

Cells were transfected with siNC or siMYH9 and were then incubated with 20 μmol/L MG132 for 0–12 h or left untreated. The cells were then treated with 50 μg/ml CHX and incubated for different periods. Cells sampled were harvested and prepared for Western blot assays.

### Statistics Analysis

Data analysis was performed using SPSS Ver.22.0 (IBM Corporation, USA) and GraphPad Prism Ver.7.0 (GraphPad Software, San Diego, CA, USA). All data are shown as the mean ± SD and were obtained from three independent experiments. Categorical variables were analyzed using Fisher's exact tests. The mean values of two groups were compared with Student's *t*-tests. Differences in survival rates among groups were estimated with Log-rank tests. Besides, the Kaplan–Meier method was used to estimate survival rate from survival curves. A two-tailed *P*-value of <0.05 was considered to be statistically significant. ^*^Indicates *P* < 0.05 and ^**^ indicates *P* < 0.01.

## Data Availability Statement

The datasets presented in this study can be found in online repositories. The names of the repository/repositories and accession number(s) can be found below:

1. RNA-SEQ: https://figshare.com/articles/RNASEQ/12210317.
2. LC/MS data: https://figshare.com/articles/mass_zip/12210314.

## Author Contributions

S-TY, J-NG, and B-HS performed *in vitro* assays. J-LS, M-SZ, and Z-GW performed *in vivo* assays. S-TY, B-HS, and T-TL collected data. S-TY and S-TL designed the study. S-TY, Z-CZ, and W-SC performed data analysis and interpretation. S-TY and B-HS designed figures. S-TY wrote this paper. All authors approved this submission.

## Conflict of Interest

The authors declare that the research was conducted in the absence of any commercial or financial relationships that could be construed as a potential conflict of interest.

## Abbreviations

PTC: papillary thyroid carcinoma
ChIP: chromatin immunoprecipitation
CHX: cycloheximide
Co-IP: co-immunoprecipitation
IHC: immunohistochemistry.
