# Supplementary figures and images for: CRLF1–MYH9 Interaction Regulates Proliferation and Metastasis of Papillary Thyroid Carcinoma Through the ERK/ETV4 Axis
Source: Front Endocrinol (Lausanne). 2020 Aug 25;11:535. doi: 10.3389/fendo.2020.00535 (PMC7477767; doi:10.3389/fendo.2020.00535)

A

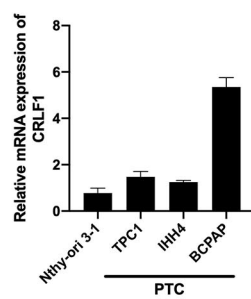

B

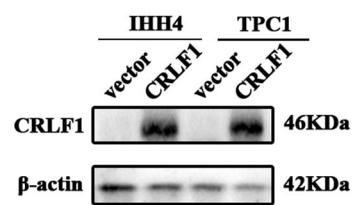

C

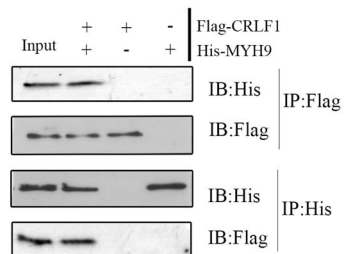

D

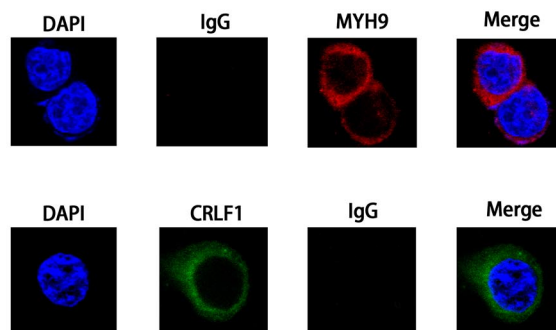

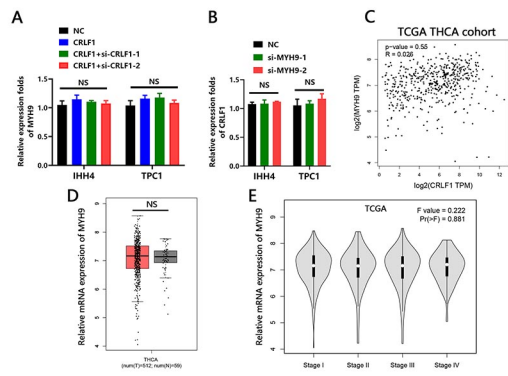

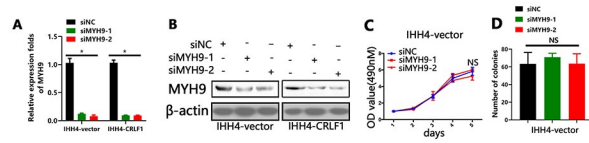

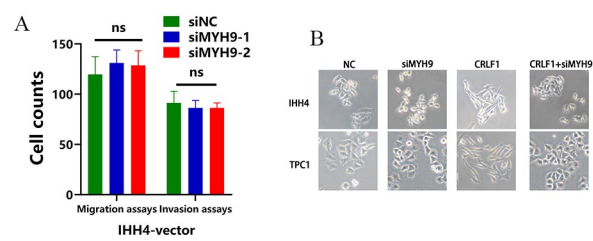

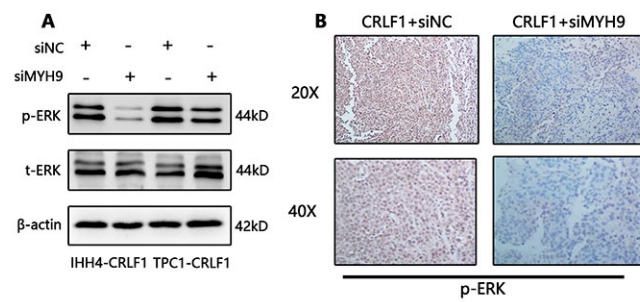

Supplement: Supplementary file 1 [file Data_Sheet_1.pdf]
