# Supplementary material for: CRLF1–MYH9 Interaction Regulates Proliferation and Metastasis of Papillary Thyroid Carcinoma Through the ERK/ETV4 Axis
Source: Front Endocrinol (Lausanne). 2020 Aug 25;11:535. doi: 10.3389/fendo.2020.00535 (PMC7477767; doi:10.3389/fendo.2020.00535)
Supplement: Supplementary file 2 [file Data_Sheet_2.pdf]

Table S1. Co-altered gene in RNA-sequence

Gene	Log2FoldChange in IHH4-V vs. IHH4-C	P value	P-adjusticed	Log2FoldChange in TPC1-V vs. TPC1-C	P value	P-adjusticed
CRLF1	6.018751891	5.81E-32	1.41E-27	2.775397653	8.87E-09	2.74E-05
ETV4	2.190044024	5.23E-07	0.00141397	2.928106106	2.65E-08	6.36E-05
TMEM238	2.202330726	7.00E-07	0.00141898	2.519164955	4.80E-06	0.00576609
ARL17B	3.45499928	1.38E-05	0.0108825	4.712097159	1.09E-05	0.01067775
TMCC2	2.149936887	1.58E-05	0.01090826	2.156383941	1.79E-05	0.01523513
STAG3L1	-3.758505596	1.34E-07	0.00026365	-2.806902903	7.89E-08	0.00017065
PKD1P1	-2.003704042	1.66E-06	0.00224671	-2.447071007	1.82E-07	0.00032873
CES1P1	-2.276710969	4.69E-06	0.00576609	-2.33811374	4.05E-07	0.00067289
GREM1	-2.05925218	1.54E-05	0.01386078	-2.008967091	1.24E-05	0.01166105

Table S2. Primers for qPCR.

CRLF1 sense	GGGATCTGGAGTGAGTGGAGC
CRLF1 anti-sense	GGGTCTTGTGCGACTTCTGC
MYH9 sense	AGTTTGTCTCGGAGCTGTGG
MYH9 anti-sense	GGTTCGTGTTCCTCAGCGTA
ETV4 sense	GATGAAAGCCGGATACTTGGAC
ETV4 anti-sense	TTCGCGCAAGCTCCCATTT
E-cadherin sense	CGAGAGCTACACGTTCACGG
E-cadherin anti-sense	GGGTGTCGAGGGAAAAATAGG
SNAI1 sense	TCGGAAGCCTAACTACAGCGA
SNAI1 anti-sense	AGATGAGCATTGGCAGCGAG
VIM sense	GACGCCATCAACACCGAGTT
VIM anti-sense	CTTTGTCGTTGGTTAGCTGGT
FN1 sense	CGGTGGCTGTCAGTCAAAG
FN1 anti-sense	AAACCTCGGCTTCCTCCATAA
MMP1 sense	AAAATTACACGCCAGATTTGCC
MMP1 anti-sense	GGTGTGACATTACTCCAGAGTTG
MMP7 sense	GAGTGAGCTACAGTGGGAACA
MMP7 anti-sense	CTATGACGCGGGAGTTTAACAT
MMP9 sense	TGTACCGCTATGGTTACACTCG
MMP9 anti-sense	GGCAGGGACAGTTGCTTCT
β-actin sense	CGCGAGAAGATGACCCAGAT
β-actin anti-sense	GGGCATACCCCTCGTAGATG

Table S3. Sequence of siRNAs

siRNA 1# of CRLF1	GGCUCUCUUACGCCCUAU
siRNA 2# of CRLF1	CACGCUGGAUAUCCUGGAU
siRNA 1# of MYH9	CCGGCAAGGTGGATTACAA
siRNA 2# of MYH9	ACACGGAGCTGATCAACGA
siRNA 1# of ETV4	GGCGCTTCCCAACTTCATA
siRNA 2# of ETV4	CCCTGTGTACATATAAATGAA
